# Identification of Potential circRNA-microRNA-mRNA Regulatory Network in Skeletal Muscle

**DOI:** 10.3389/fmolb.2021.762185

**Published:** 2021-11-29

**Authors:** Arundhati Das, Sharmishtha Shyamal, Tanvi Sinha, Smruti Sambhav Mishra, Amaresh C. Panda

**Affiliations:** ^1^ Institute of Life Sciences, Nalco Square, Bhubaneswar, India; ^2^ School of Biotechnology, KIIT University, Bhubaneswar, India

**Keywords:** circular RNA, microRNA, skeletal muscle, myogenesis, ceRNA

## Abstract

Circular RNAs (circRNAs) are a newly discovered family of regulatory RNAs generated through backsplicing. Genome-wide profiling of circRNAs found that circRNAs are ubiquitously expressed and regulate gene expression by acting as a sponge for RNA-binding proteins (RBPs) and microRNAs (miRNAs). To identify circRNAs expressed in mouse skeletal muscle, we performed high-throughput RNA-sequencing of circRNA-enriched gastrocnemius muscle RNA samples, which identified more than 1,200 circRNAs. In addition, we have identified more than 14,000 and 15,000 circRNAs in aging human skeletal muscle tissue and satellite cells, respectively. A subset of abundant circRNAs was analyzed by RT-PCR, Sanger sequencing, and RNase R digestion assays to validate their expression in mouse skeletal muscle tissues. Analysis of the circRNA-miRNA-mRNA regulatory network revealed that conserved *circNfix* might associate with miR-204-5p, a suppressor of myocyte enhancer factor 2c (*Mef2c*) expression. To support the hypothesis that *circNfix* might regulate myogenesis by controlling *Mef2c* expression, silencing *circNfix* moderately reduced *Mef2c* mRNA expression and inhibited C2C12 differentiation. We propose that *circNfix* promotes *MEF2C* expression during muscle cell differentiation in part by acting as a sponge for miR-204-5p.

## Introduction

Skeletal muscle is the largest organ contributing to one-third of the human body weight. Skeletal muscle is responsible for voluntary movement and has a high degree of regeneration ability. The fusion of the myoblasts generates the multinucleated long muscle fibers with contractile properties, and the process is known as myogenesis (Okazaki and Holtzer, 1966). The expression of muscle-specific transcription factors such as MyoD, Myog, Myf5, and Mef2a-d at various points collaboratively regulate multiple muscle formation steps, including myoblast proliferation, cell cycle exit, muscle-specific gene expression, myoblast fusion, and sarcomere assembly (Buckingham and Rigby, 2014). In addition to the well-known myogenic transcription factors, noncoding RNAs such as microRNAs, lncRNAs, and the recently discovered circular RNAs (circRNAs) were also involved in muscle cell differentiation and muscle development (Zhao et al., 2019; Das et al., 2020).

CircRNAs are one class of closed-loop single-stranded RNA molecules generated by the head-to-tail splicing of pre-mRNA (Chen and Yang, 2015). Due to the closed circular nature, circRNAs are resistant to exonucleases and very stable compared to linear RNAs. circRNAs have been reported to regulate gene expression by acting as sponges for RNA-binding proteins (RBPs) and miRNAs (Panda, 2018; Das et al., 2021). Also, a few circRNAs are translated into polypeptides with the cap-independent translation mechanisms, and some circRNAs regulate transcription by associating with snRNA and RNA pol II (Bose and Ain, 2018; Sinha et al., 2021). CircRNAs are dynamically expressed and involved in various physiological processes, including development, differentiation, and aging (Abdelmohsen et al., 2015; Di Timoteo et al., 2020). Interestingly, global upregulation of circRNAs was observed in the aged brain compared to young mice and *drosophila* (Westholm et al., 2014; Gruner et al., 2016). Although recent studies underscored the importance of circRNAs in muscle physiology, the expression and functions of circRNAs in muscle regeneration are still not understood completely.

This study sought to characterize circRNAs expressed in mouse skeletal muscle. Here, we identified circRNAs expressed in young and aged skeletal muscles. We also predicted their association with muscle miRNAs and downstream target genes. The circRNA-miRNA-mRNA regulatory network highlighted their possible role in muscle physiology**.** Also, we analyzed the previously published RNA-sequencing data from young and old human skeletal muscle and satellite cells to identify age-associated circRNAs. Together, our study provides new information to better understand the age-associated decline in muscle function and develop a new therapeutic strategy for age-associated muscle diseases.

## Methods

### Animals and Sampling

Young (6-weeks old) and aged (17-months old) male BALB/c mice were acquired from the Institute of Life Sciences breeding colonies. All animals were raised under standard conditions. The gastrocnemius muscle was collected from each mouse, washed in ice-cold PBS, and lysed in TRIzol for RNA isolation immediately or stored in −80°C for further use. All experimental procedures were conducted according to the approved guidelines of the institutional animal ethics committee of the Institute of Life Sciences.

### Muscle Cell Differentiation, Circular RNA Silencing, and Immunostaining

High glucose Dulbecco’s modified Eagle’s medium (DMEM) supplemented with 15% FBS and antibiotics was used to culture the C2C12 mouse myoblast cell line in 5% CO_2_ at 37°C (Pandey et al., 2020). Human skeletal muscle myoblasts (HSMM) cells (Lonza) were cultured in Skeletal Muscle Cell Growth Medium-2 Bullet Kit (Lonza, CC-3245) and subcultured using subculture reagents (Lonza, CC-5034) following the instructions. The growth media of the sub-confluent C2C12 and HSMM cells were replaced with the differentiation media (DMEM supplemented with 2% horse serum), and the cells were differentiated into myotubes for up to 4 days. The C2C12 and HSMM differentiation was monitored with a phase-contrast microscope to check the formation of elongated multinucleated myotubes. The differentiated HSMM myotubes were immunostained with Anti-heavy chain Myosin/MYH3 antibody (Abcam# ab124205) followed by Goat Anti-rabbit IgG H&L (Alexa Fluor^®^ 488) fluorescent secondary antibody against MYH3 antibody (Abcam #ab150077) and DAPI (Sigma #D9542) for nuclear staining. For *circNfix* silencing experiments, the C2C12 cells were transfected with control GapmeR (ctrl GapmeR) or circNfix GapmeR twice (36 and 12 h before inducing differentiation) using Lipofectamine RNAiMAX transfection reagent (Invitrogen) following manufacturer’s instructions. The transfected cells were allowed to differentiate for 4 days before using them for differentiation analysis. The 4-day differentiated cells were stained with Jenner–Giemsa stains to analyze the differentiation efficiency as described previously (Velica and Bunce, 2011).

### RNA Sequencing and Circular RNA Analysis

The total RNA from two young and two aged gastrocnemius muscles was prepared using TRIzol (Thermo). Seven μg of total RNA was treated with RNase R to enrich the circRNA population. A total of 500 ng RNase R treated RNA was fragmented, and the cDNA library was prepared using the NEBNext^®^ Ultra™ II Directional RNA Library Prep Kit for Illumina, following the manufacturer’s instructions. The cDNA library quantity and quality was analyzed using Qubit and TapeStation, respectively. The libraries were sequenced for 75 bp paired-end reads on the Illumina NextSeq 550 platform using NextSeq 550 High Output Kit v2 (Illumina TG-160–2002); data are deposited in ENA (accession number PRJEB46548). The two young samples were sequenced with 53 and 62 million reads, while the aged samples were sequenced with 40 and 55 million reads. The adapter contamination was removed from the raw fastq files, then aligned to the mouse genome (mm10) using the STAR aligner using ChimSegmentMin-10 parameter. CIRCexplorer2 (v2.3.6) pipeline was used to identify the circRNAs (Zhang et al., 2016). Since circRNA read numbers were low for most circRNAs, we used transcripts per million (TPM) as normalized circRNA expression levels using the formula (circRNA read number/total reads in the sample x 1,000,000). The circRNA backsplice junction read numbers from two young and two aged muscle samples are provided in Supplementary Table S1.

Previously published total RNA-seq data of human skeletal muscle samples from young (22–35 years) and aged (70–82) were downloaded from NCBI GEO (Tumasian et al., 2021) (GEO accession: GSE164471; SRA ID: SRP300916) (Supplementary Table S2). In addition, total RNA-seq data of human muscle satellite cells from young and aged donors were obtained from NCBI GEO (GEO Accession No. GSE78611) using the SRA toolkit (v2.10.0). SRA data was converted into fastq data for further analysis. FastQC software (v0.11.2) was used to assess the sequencing quality of raw data in fastq format. The circRNA expression in these human muscle tissue and satellite cells was identified using CIRCexplorer2 (Zhang et al., 2016). The normalized circRNA expression in each samples of human muscle tissue and satellite cells were obtained using TPM calculation as described above (Supplementary Table S3, S4). A subset of abundant circRNAs with higher read numbers in the RNA-seq analysis and with length <2 Kb was selected for further analysis.

### RT-PCR, Sanger Sequencing, RNase R Treatment, and QuantitativePCR

Total RNA from gastrocnemius muscle were isolated using TRIzol or Total RNA isolation kit (HiMedia) followed by cDNA synthesis using High Capacity cDNA Reverse Transcription kit following the manufacturer’s protocol (Thermo Fisher Scientific). The target circRNAs were PCR amplified with RNA-specific primers using the following program: denaturation at 95°C for 2 min, followed by 40 cycles of denaturation at 95°C for 5 s, annealing, and extension at 60°C for 20 s (Supplementary Table S5) (Panda and Gorospe, 2018). The PCR amplicons were resolved on a 2% agarose gel stained with SYBR-Gold and visualized on an ultraviolet transilluminator. The circRNA backsplice junction sequences are confirmed by Sanger sequencing of the PCR products amplified with the divergent primers. Quantitative (q)PCR analysis of target RNAs was performed using target-specific primers and 2X PowerUP SYBR Green Master Mix (Thermo Fisher Scientific). The relative expression levels of the target RNA were calculated using the delta-CT method considering 18S rRNA or Gapdh mRNA as internal controls. Total RNA from C2C12 cells was treated with RNase R enzyme (Epicentre) followed by RT-qPCR analysis to test the circular nature of selected circRNAs (Panda and Gorospe, 2018).

Total RNA was reverse transcribed with miR-X first-strand synthesis kit following the manufacturer’s protocol (Takara). The cDNA was diluted, and RT-qPCR was performed using diluted cDNA, PowerUP SYBR Green Master Mix, and the specific forward primers for the miRNAs with the common reverse primer provided with the miR-X first-strand synthesis kit. The relative miRNA expression was measured with the delta-CT method using U6 as the endogenous loading control (Livak and Schmittgen, 2001).

### Identification of circRNA-miRNA-mRNA Interactions

The spliced sequences of the selected circRNAs were provided as input in the custom prediction tool of the miRDB web tool and TargetScan software (release 7.2) to identify circRNA-associated miRNAs (Agarwal et al., 2015; Chen and Wang, 2020). The list of miRNAs expressed in mouse skeletal muscle was derived from a previous publication (GEO accession ID: GSE55164) (Kim et al., 2014). The miRNAs expressed in mouse skeletal muscle and the validated target genes in the miRTarBase release 8.0 were considered for further analysis (Huang et al., 2020) (Supplementary Table S6).

### Gene Ontology and Kyoto Encyclopedia of Genes and Genomes Pathway Analysis

The functions of circRNAs were determined by the genes targeted in the circRNA-miRNA-mRNA regulatory network. The STRING database (v11.5) was used for comprehensive analysis for the target genes using GO (http://www.geneontology.org/) and the KEGG (https://www.kegg.jp/) to understand the biological functions (Supplementary Table S7) (Kanehisa et al., 2016; The Gene Ontology, 2019; Szklarczyk et al., 2021). The GO enrichment analysis of the target genes found several significantly enriched (False Discovery Rate: FDR, *p*-value<0.05) GO terms, including Biological Process (BP), Molecular Function (MF), and Cellular Component (CC). The enrichment score for the GO terms and KEGG pathways were calculated as Log10 of the FDR value and plotted as bubble plots.

### Construction of the Competing Endogenous RNA (ceRNA) Network and Visualization

GraphPad Prism 6.0 software, R studio or Microsoft excel was used to plot the graphs for data visualization. We used Cytoscape software (v3.8.2) to construct and visualize the circRNA-miRNA-mRNA network in this study. The bubble plots were generated using ggplot2 in R studio. Statistical significance was calculated by student’s t-test and considered significant with a *p*-value of <0.05.

## Results

### Characteristics of circRNAs in Mouse Skeletal Muscle Tissue

**FIGURE 1:**
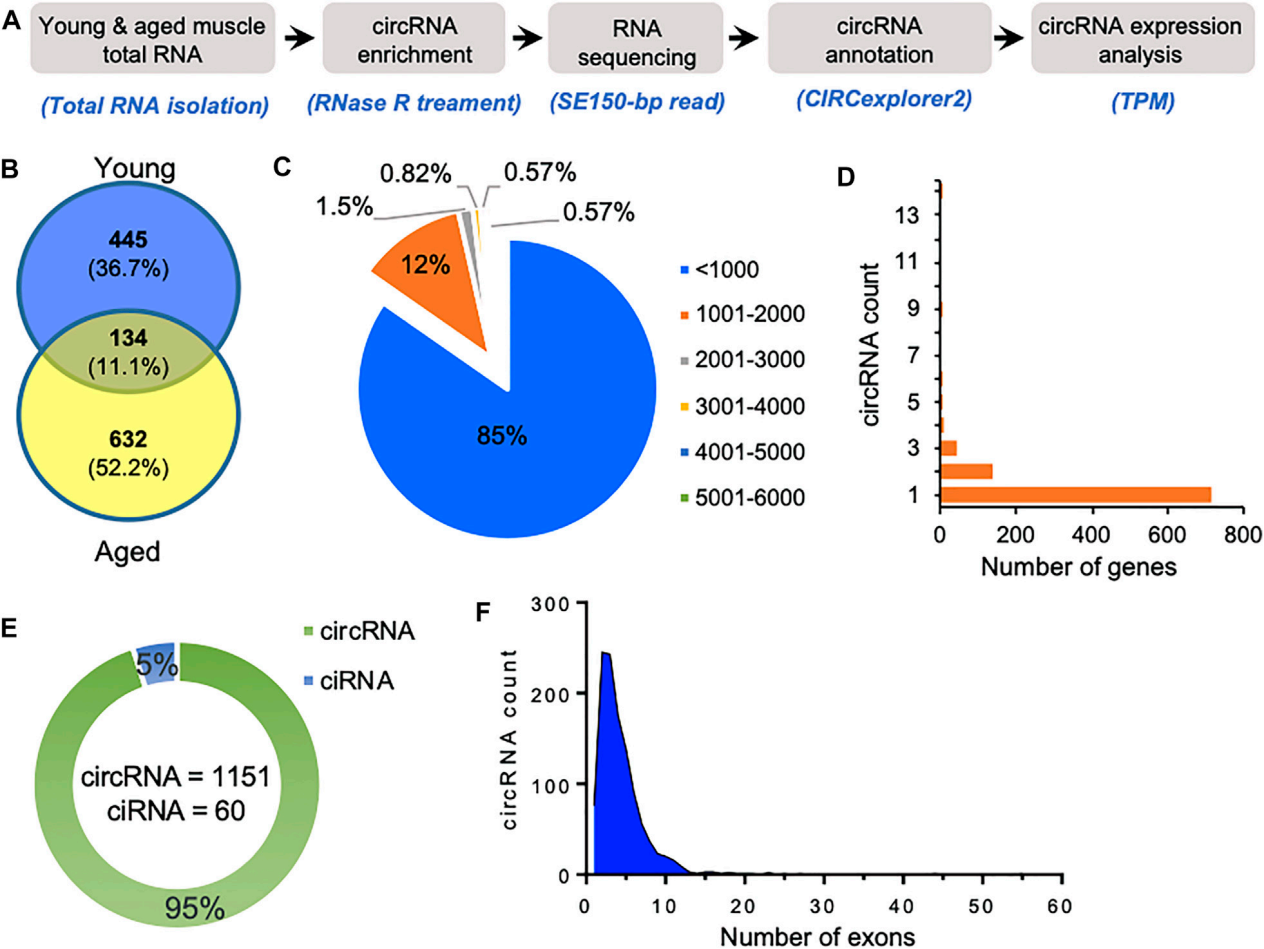
Characteristics of circRNAs expressed in aging mice skeletal muscle. **(A)**. The experimental design and annotation of circRNAs using CIRCexplorer2 in mouse gastrocnemius muscle. **(B)**. Venn diagram showing the number of circRNAs expressed in young and aged gastrocnemius muscle identified using CIRCexplorer2. **(C)**. Percentage distribution of length of circRNAs in mouse skeletal muscle. **(D)**. Number of genes corresponding to the number of circRNAs generated by them. **(E)**. Number of exonic circRNA and intronic ciRNAs expressed in mouse skeletal muscle. **(F)**. Distribution of the number of exons in the exonic circRNAs expressed in gastrocnemius muscle detected with CIRCexplorer2.

We used gastrocnemius skeletal muscle of young and aged mice for the identification of skeletal muscle circRNAs. As shown in Figure 1A, RNA-seq libraries were prepared from circRNA enriched RNA samples followed by sequencing. The RNA-seq reads were further processed to identify circRNAs using CIRCexplorer2 (Zhang et al., 2016). Interestingly, the aged samples contained a higher percentage of chimeric reads and non-canonical splice junctions. Moreover, the total number of circRNAs identified in aged skeletal muscle was significantly higher than in young samples (Figure 1B). Consistent with previous reports, our data suggest an overall upregulation and accumulation of circRNAs in aged skeletal muscle (Gruner et al., 2016).

As shown in Figure 1C, most of the identified circRNAs were smaller than 1,000 nucleotides in length. Interestingly, most circRNA host genes generated only one or two circRNAs, while a few host genes generated multiple circRNAs (Figure 1D). Since circRNAs can be generated from the exonic and intronic sequences, our circRNA annotation suggested that ∼95% of circRNAs were exonic circRNAs while a few were intronic ciRNAs (Figure 1E). Analysis of the number of exons in the exonic circRNAs revealed that most circRNAs harbor less than ten exons (Figure 1F). The expression of circRNAs in young and aged skeletal muscle was performed using the TPM values. The complete list of the 1,211 circRNAs identified in aging mouse gastrocnemius muscle is provided (Supplementary Table S1), including circRNA annotation, chromosomal coordinates, length, and expression values. Moreover, only a few circRNAs were differentially expressed, and most of the circRNAs identified in mice skeletal muscle samples were expressed at a very low level. The low abundance and small number of circRNAs in the sequencing data could be due to fewer replicates, low depth, and smaller read-length in RNA sequencing.

Since circRNAs are known to be conserved across various species, we sought to identify circRNAs expressed in aging human skeletal muscle and muscle satellite cells. To identify circRNAs in human skeletal muscle, previously published total RNA-seq data from young (22–35 years) and aged (70–82 years) human skeletal muscle RNA-seq data were analyzed using CIRCexplorer2 pipeline (GEO ID: GSE164471) (Supplementary Table S3). We have identified more than 14,000 circRNAs, most derived from exonic sequences and less than 1,000 nucleotides in length (Supplementary Figure S1A–C). Moreover, most of the genes produced a few circRNAs, while a few genes such as TTN and NEB generate hundreds of circRNAs (Supplementary Figure S1D,E). Furthermore, we also analyzed the circRNAs expressed in young and aged muscle satellite cells (Supplementary Table S4, Supplementary Figure S2). We also identified more than 15,000 circRNAs, where 90% are exonic circRNAs, and the rest are intronic ciRNAs (Supplementary Figure S2).

### Validation of circRNAs in Mice Skeletal Muscle

**FIGURE 2:**
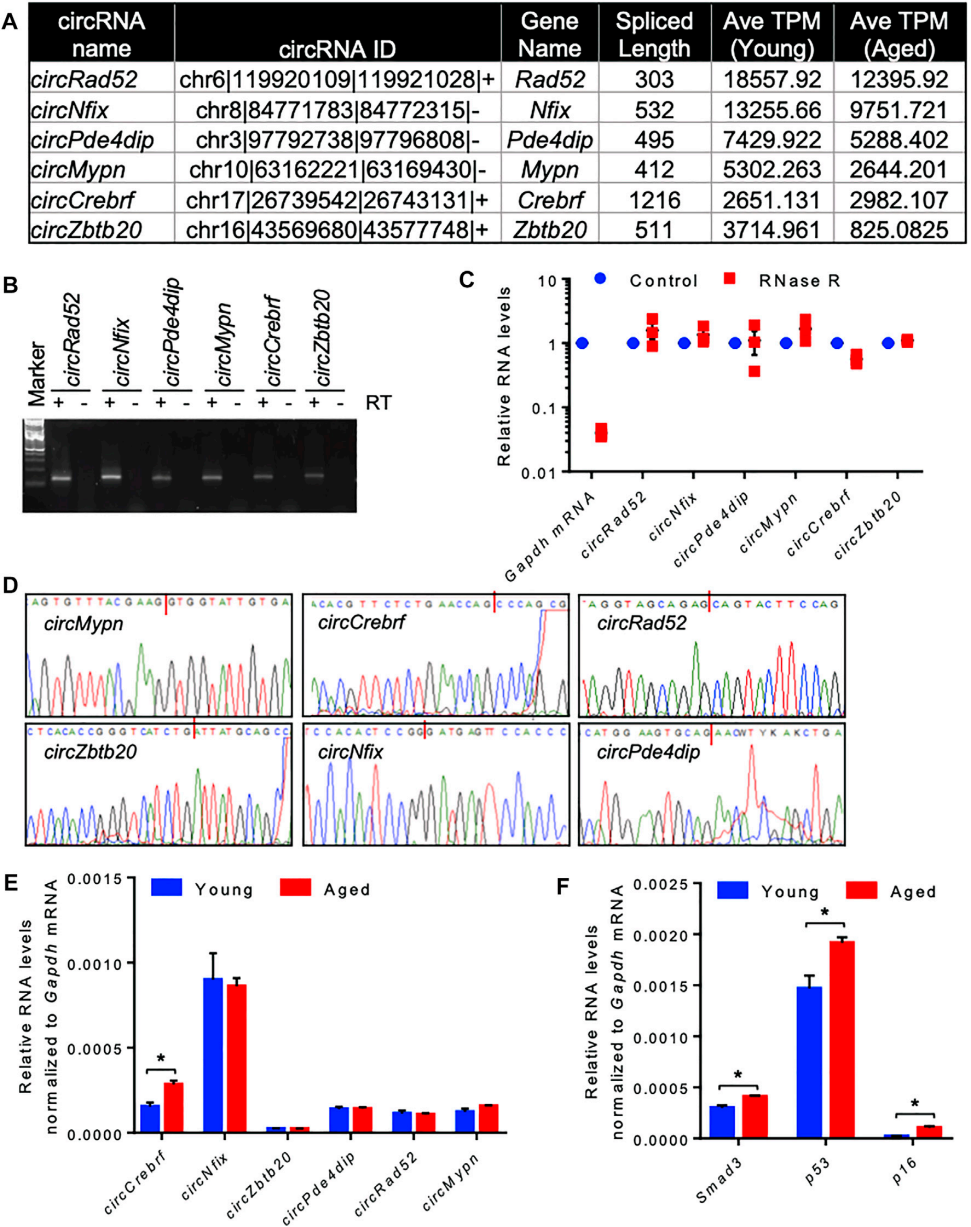
Differential expression and validation of circRNAs in aging skeletal muscle. **(A)**. A list of abundant circRNAs was selected for further validation. **(B)**. The RT-PCR products of circRNAs from muscle tissue were resolved on a 2% agarose gel stained with SYBR Gold. **(C)**. RT-PCR analysis of circRNAs and linear RNAs upon RNase R treatment. **(D)**. PCR products of circRNAs from muscle were purified and sequenced to confirm the backsplice sequences. **(E,F)**. RT-qPCR analysis of circRNAs (E) and mRNAs (F) in young and aged skeletal muscle normalized to *Gapdh* mRNA. Data in (C,E,F) are the means ± SEM from three independent experiments. *, *p* < 0.05.

Since most of the circRNAs were very low abundant in the RNA-seq data, we selected a few highly expressed circRNAs for differential expression analysis using quantitative RT-PCR (Figure 2A). Using divergent primers, RT-PCR amplification of the circRNAs specifically amplified the target circRNAs without amplifying the no-RT control (Supplementary Table S5, Figure 2B). We also performed RNase R exonuclease treatment followed by RT-qPCR to analyze the circular nature of the circRNAs. The linear *Gapdh* mRNA was degraded to minimal levels while all tested circRNAs were resistant to RNase R digestion, confirming the circular nature of the circRNAs (Figure 2C). Furthermore, Sanger sequencing of the PCR products confirmed the amplification of the backsplice junction of the target circRNAs (Figure 2D, Supplementary Figure S3). Among the tested circRNAs, *circCrebrf* was moderately upregulated along with elevated expression of age-associated *p53, p16*, and *Smad3* mRNAs in the aged muscle samples compared to the young muscle (Figures 2E,F).

### Prediction of Potential circRNA-miRNA-mRNA Regulatory Axis in Skeletal Muscle

**FIGURE 3:**
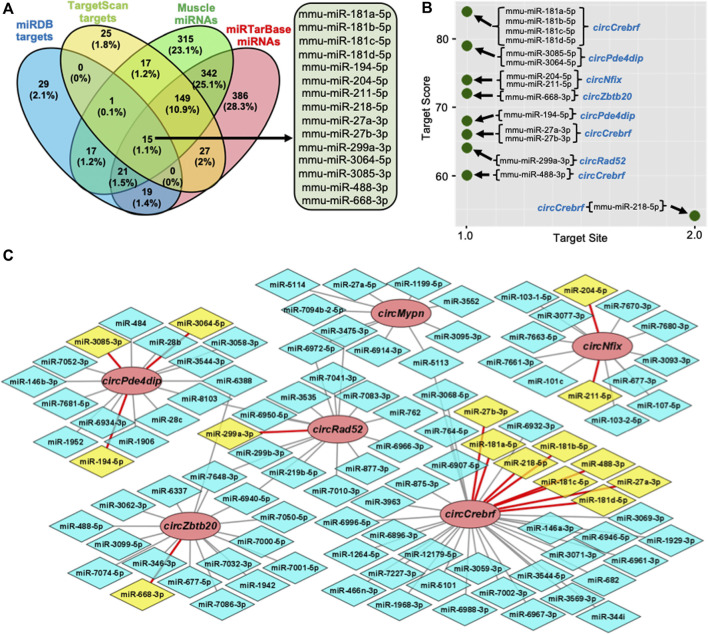
Prediction of the circRNA-miRNA-mRNA regulatory network in skeletal muscle. **(A)**. Venn diagram depiction of the muscle miRNAs predicted to target the validated circRNAs and validated by miRTarBase. **(B)**. Predicted miRNA targets of the six validated circRNAs using miRDB custom prediction tool. **(C)**. The circRNA-miRNA regulatory network was constructed and visualized on Cytoscape. Circle nodes represent circRNAs, hexagon nodes represent miRNAs, and yellow miRNAs with red edges are functional miRNAs.

Recent studies established that circRNAs function as inhibitors of circRNA-interacting miRNAs, thereby regulating the target gene expression. To identify the circRNA associated miRNAs, the circRNA sequences were used to predict the target miRNAs using miRDB and TargetScan software (Agarwal et al., 2015; Chen and Wang, 2020). Computational prediction identified 102 potential miRNAs targeting the six validated circRNAs using miRDB, and 234 miRNAs were identified with TargetScan (Supplementary Table S6). The previous publication reported the expression of 877 miRNAs in mouse gastrocnemius muscle (Supplementary Table S6) (Kim et al., 2014). To find the functional miRNA in skeletal muscle targeted by these circRNAs, we identified the common muscle miRNAs targeted by these six circRNAs and have experimentally validated mRNA targets reported by miRTarBase (Figure 3A; Supplementary Table S6) (Huang et al., 2020). This analysis identified fifteen functional circRNA-associated miRNAs, including mouse miR-181a-5p, miR-181b-5p, miR-181c-5p, miR-181d-5p, miR-194-5p, miR-204-5p, miR-211-5p, miR-218-5p, miR-27a-3p, miR-27b-3p, miR-299a-3p, miR-3064-5p, miR-3085-3p, miR-488-3p, and miR-668-3p (Figure 3A). Interestingly, these fifteen miRNAs were associated with five out of six circRNAs (Figures 3B,C). Analysis of the downstream genes for these miRNAs using the miRTarBase 8.0 database revealed that these fifteen miRNAs have 866 validated mRNA targets (Supplementary Table S6).

To find the functional significance of the downstream target genes in the circRNA-miRNA-mRNA regulatory network, we performed GO enrichment analysis for the biological process, cellular component, and molecular function (Supplementary Table S7). As shown in Supplementary Figure S4, several GO terms are enriched for the target genes of circRNAs. Briefly, multicellular organism development, cellular process, developmental process, anatomical structure development, and system development were the top GO biological processes, whereas binding, protein binding, ion binding, and organic cyclic compound binding were among the top hits in the GO molecular functions, and cell, intracellular, cytoplasm, intracellular organelle, organelle, and membrane-bounded organelle were among the top GO cellular component terms. Furthermore, KEGG pathway enrichment analysis of circRNA target genes identified several pathways critical for cell survival and aging (Supplementary Figure S4A–C; Supplementary Table S7). The common pathways with higher enrichment scores, include axon guidance, microRNAs in cancer, hippo signaling pathway, AGE-RAGE signaling pathway in diabetic complications, pathways in cancer, HIF-1 signaling pathway, and FoxO signaling pathway (Supplementary Figure S4D).

### Conserved *circNfix* and Its Regulatory Role in Muscle Cell Differentiation

Since circRNAs are conserved across various species, we wanted to find the conserved circRNAs in mouse and human skeletal muscles (Ji et al., 2019). To identify the conserved mouse muscle circRNAs, we converted the mouse muscle circRNA coordinates to hg38 human coordinates using the LiftOver tool (Haeussler et al., 2019). Analysis of the conservation of the circRNAs expressed in mouse skeletal muscle and human muscle or satellite cells identified several conserved circRNAs (Supplementary Figure S5A). Of the six validated circRNAs, *circMypn* was conserved in human skeletal muscle and satellite cells, while *circNfix* was conserved in human satellite cells. Consistent with our mouse skeletal muscle data, *circNFIX* was upregulated in aged satellite cells compared to young satellite cells (Supplementary Figure S5B). The NCBI BLAST analysis suggested that the human *circNFIX* sequence is 96% similar to mouse *circNfix*, indicating conserved in humans and mice (Supplementary Figure S5C,D). In addition, the circRNA database circBase reported the expression of *circNFIX* (*hsa_circ_0005660*) in HSMM cells (Glazar et al., 2014). Since *circMypn* did not target any functional muscle miRNAs and the differentially expressed *circCrebrf* was not found to be conserved in human muscle, we wanted to analyze the role of *circNfix* in muscle.

### Conserved *circNfix* Is Required for Efficient Myogenesis

**FIGURE 4:**
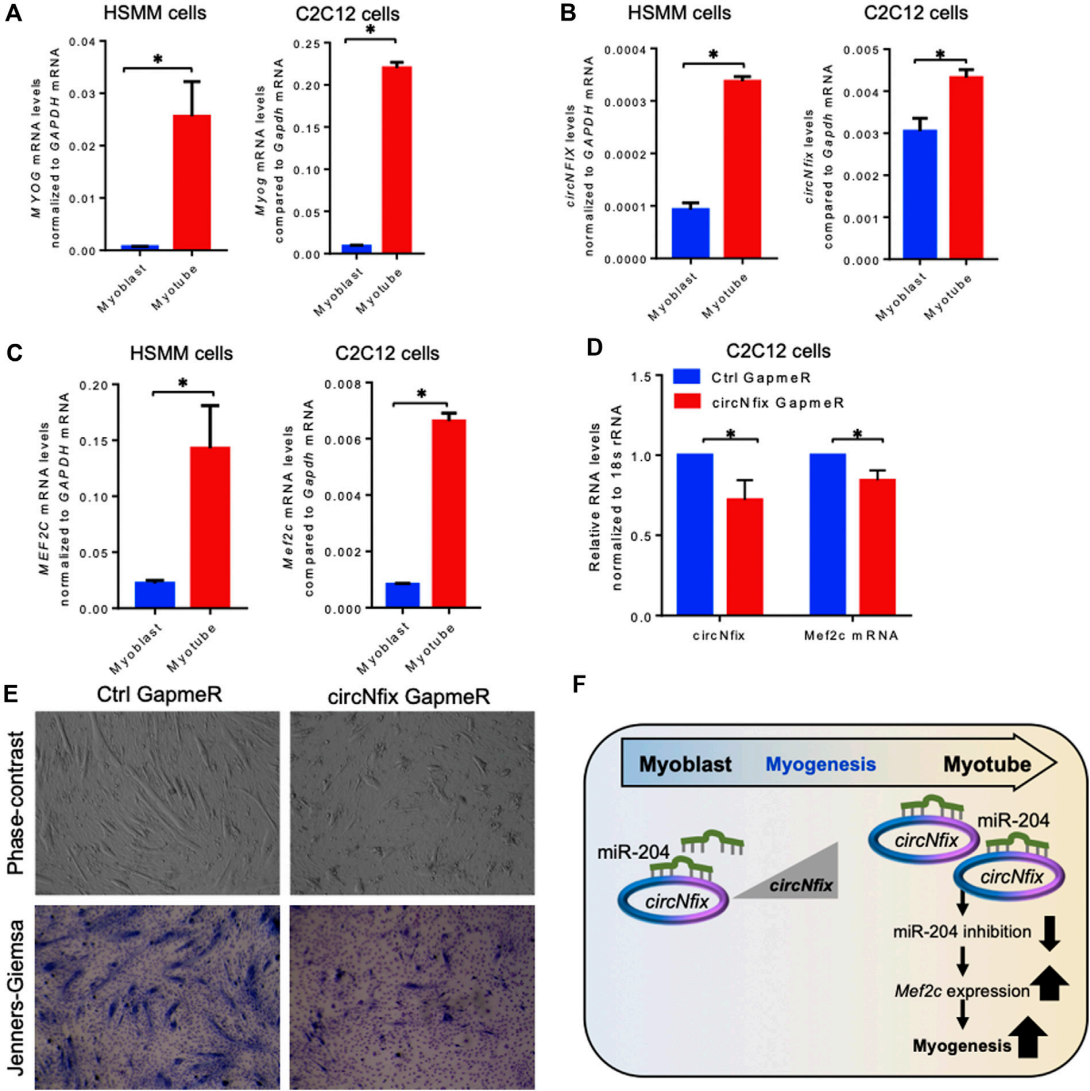
Expression analysis of circNFIX and downstream targets. **(A)**. Relative expression of *MYOG* mRNAs in proliferating and 4-day differentiated myotubes of HSMM and C2C12 cells. **(B)**. Expression levels of *circNFIX* measured by RT-qPCR analysis in proliferating myoblasts and 4-day differentiated myotubes of HSMM and C2C12 cells. **(C)**. RT-qPCR analysis of *MEF2C* mRNA levels in proliferating myoblasts and 4-day differentiated myotubes of HSMM and C2C12 cells. **(D)**. RT-qPCR analysis of circNfix and Mef2c expression levels in 4 day differentiated C2C12 cells transfected with Ctrl and circNfix GapmeR 24 hours before starting differentiation. **(E)**. The phase-contrast fields and Jenners-Giemsa stained 4-day differentiated C2C12 cells transfected with Ctrl and circNfix GapmeR 24 hours before starting differentiation. **(F)**. Proposed hypothesis of *circNfix* action during myoblast differentiation. Data in (A,B,C,D) are the means ± SEM from three independent experiments. *, *p* < 0.05.

It has been established that circRNAs act as competing endogenous RNAs (ceRNA) for target miRNAs, and circRNA expression changes positively correlate with the downstream target mRNAs. As shown in Figure 3C, *circNfix* is predicted to associate with many miRNAs, including miR-204-5p, that regulate myogenesis. The *circNfix*-associated miR-204 is known to inhibit myogenesis by suppressing the Mef2c expression in C2C12 cells (Cheng et al., 2018). Analysis of *circNfix-*associated miR-204 and the downstream myogenic regulator *Mef2c* mRNA in aged gastrocnemius muscle did not show any changes (Supplementary Figure S6A,B). To analyze the expression and function of conserved *circNFIX/circNfix* in human and mouse skeletal muscle, we differentiated sub-confluent HSMM and C2C12 cells for 4 days in differentiation media to induce the myotube formation. As shown in Figure 4A, the HSMM and C2C12 cells form long myotubes, and the differentiation was confirmed by upregulation of myogenic marker *MYOG* mRNA by qPCR analysis (Figure 4A, Supplementary Figure S7A). We further confirmed the expression of *circNFIX* in HSMM cells by RT-PCR followed by Sanger Sequencing (Supplementary Figure S7B). Interestingly, *circNFIX* expression was significantly upregulated in 4-day differentiated HSMM and C2C12 myotubes than proliferating myoblasts (Figure 4B). However, miR-204-5p levels did not alter significantly in 4-day differentiated HSMM and C2C12 myotubes compared to proliferating myoblasts (Supplementary Figure S7C,D). Notably, *MEF2C* mRNA, the downstream target of *circNFIX,* was significantly upregulated in 4-day differentiated HSMM and C2C12 myotubes supporting the circRNA ceRNA regulatory network hypothesis (Figure 4C).

Given that miR-204 inhibits myogenesis by suppressing MEF2C expression (Cheng et al., 2018), we hypothesized that *circNfix* might inhibit miR-204 activity to promote MEF2C expression and C2C12 differentiation into myotubes. Interestingly, silencing *circNfix* reduced *Mef2c* mRNA expression moderately without significant changes in miR-204 levels in the 4-day differentiated C2C12 cells (Figure 4D, Supplementary Figure S7E). These results suggested that *circNfix* might control the expression of *Mef2c* mRNA by sponging miR-204. However, the antisense oligo pulldown assay of *circNfix* using a biotin-labeled oligo targeting the backsplice junction could not pulldown *circNfix* (data not shown), which could be due to the unavailability of the circRNA junction that is either hidden by associated RBPs or due to secondary structures. Analysis of myogenesis using phase-contrast and Jenners-Giemsa staining followed by microscopy showed that silencing of *circNfix* decreased myogenesis and myotube formation in 4-day differentiated C2C12 cells (Figure 4E). Although *Mef2c* mRNA is known to be upregulated during muscle cell differentiation through transcriptional mechanisms, our results suggest that upregulation of *circNfix* in differentiated myotubes may partly enhance *MEF2C* expression by inhibiting the activity of miR-204 (Figure 4F). However, further experiments are required to establish the direct interaction and the circNfix-miR-204-MEF2C regulatory axis modulating myogenesis.

## Discussions

CircRNAs are a new class of universally expressed single-stranded RNA molecules (Salzman et al., 2012; Jeck et al., 2013). Recent studies suggested that circRNAs are conserved across various species and show tissue-specific expression (Jeck et al., 2013; Ji et al., 2019). Due to their circular nature and lack of free ends, circRNAs are highly stable and resistant to cellular exonucleases (Suzuki et al., 2006; Enuka et al., 2016). It has been well established that circRNAs associate with cellular regulators and modulate cellular events by controlling transcription, splicing, mRNA stability, and translation (Vromman et al., 2021). Several studies highlighted the role of circRNAs in various diseases and normal physiological processes, including aging (Abdelmohsen et al., 2015; Cai et al., 2019). However, the role of circRNAs in skeletal muscle regeneration and myogenesis has not been well understood yet.

This study presents a comprehensive analysis of circRNAs expressed in aging mouse skeletal muscle tissues (Figure 1). Previous studies suggested an accumulation of circRNAs with advancing age (Gruner et al., 2016). Consistent with previous study, we also observed that circRNA expressions in the aged skeletal muscle were significantly higher than in the young muscle samples due to age-associated circRNA upregulation or accumulation. Since circRNAs act as miRNA sponges to regulate the downstream gene expression, we analyzed the association of circRNAs with functional miRNAs expressed in muscle (Panda, 2018). Computational analysis of the target genes of circRNA-associated miRNAs suggested their involvement in various biological processes and pathways involved in muscle cell physiology (Supplementary Figure S4).

Since circRNAs are known to be conserved, we analyzed the conservation of the six validated mouse circRNAs in the human skeletal muscle and satellite cell. Interestingly, *circMypn* was expressed in all three data sets, while *circNfix* was expressed in satellite cells and upregulated in aged muscle satellite cells. To explore the function of conserved *circNfix*, we used the C2C12 and HSMM myoblast differentiation models. Previous publications reported that miR-204 inhibits MEF2C expression and regulates myogenesis (Cheng et al., 2018; Tan et al., 2020). Our analysis suggested an increase in *circNfix/circNFIX* expression in the differentiated C2C12 and HSMM myotubes compared to myoblasts. Interestingly, the miR-204 levels in C2C12 and HSMM cells did not alter significantly after 4 days of differentiation. Moreover, silencing *circNfix* decreased the expression of downstream *Mef2c* mRNA without changes in target miR-204 supporting the ceRNA regulatory network (Figure 4). Furthermore, silencing *circNfix* inhibited C2C12 myotube formation suggesting that *circNfix* is a positive regulator of myogenesis. We propose that increased *circNfix* levels during myogenesis may inhibit miR-204 activity, promoting *MEF2C* expression in the myotubes. We acknowledge that *MEF2C* expression is upregulated at the transcriptional level during muscle cell differentiation (Buckingham and Rigby, 2014). Since *MEF2C* expression is crucial for forming functional myotubes, *circNfix* helps in the higher expression of MEF2C by inhibiting the activity of the miR-204 during differentiation. In sum, we propose that upregulation of *circNfix* during myogenesis favors myotube formation by enhancing MEF2C expression by acting as a sponge for miR-204.

Although our data highlights the importance of circRNAs in myogenesis, further experiments are required to support the findings. All the circRNAs and their target miRNA or mRNAs were predicted computationally, which need to be experimentally validated. Further experiments are required to validate the differential expression of all the validated circRNAs and downstream miRNAs or mRNAs in aging skeletal muscle and myogenesis cell line models. It will be essential to perform pulldown and reporter assays to confirm the direct interaction of *circNfix* with miR-204. In addition, biochemical experiments are required to confirm the circNfix-miR-204-*Mef2c* regulatory axis that modulates skeletal muscle differentiation. Furthermore, the regulation of myogenesis by *circNfix* through other mechanisms such as sponging other miRNAs or RBPs remains to be studied.

In summary, we generated a comprehensive genome-wide circRNA expression profile of 1,200 circRNAs in mouse skeletal muscle. In addition, we have identified more than 14,000 circRNAs in human skeletal muscle and human muscle satellite cells. We have identified the ceRNA network for a subset of abundantly expressed circRNAs and their regulatory networks in skeletal muscle. Finally, we identified *circNfix* as a promoter of myogenesis by acting as a sponge for miR-204. Although our study provides new insight into the skeletal muscle gene regulation by circRNAs, further research is warranted to discover novel therapeutic targets to better manage muscle diseases and muscle regeneration.

## Data Availability

All data generated in this study are included in the main text or the supplementary information files. The raw reads produced in this study were deposited in the European Nucleotide Archive (ENA accession number PRJEB46548).
