# Supplementary material for: Identification of Potential circRNA-microRNA-mRNA Regulatory Network in Skeletal Muscle
Source: Front Mol Biosci. 2021 Nov 29;8:762185. doi: 10.3389/fmolb.2021.762185 (PMC8666571; doi:10.3389/fmolb.2021.762185)
Supplement: Supplementary file 4 [file DataSheet1.PDF]

Supplementary Material

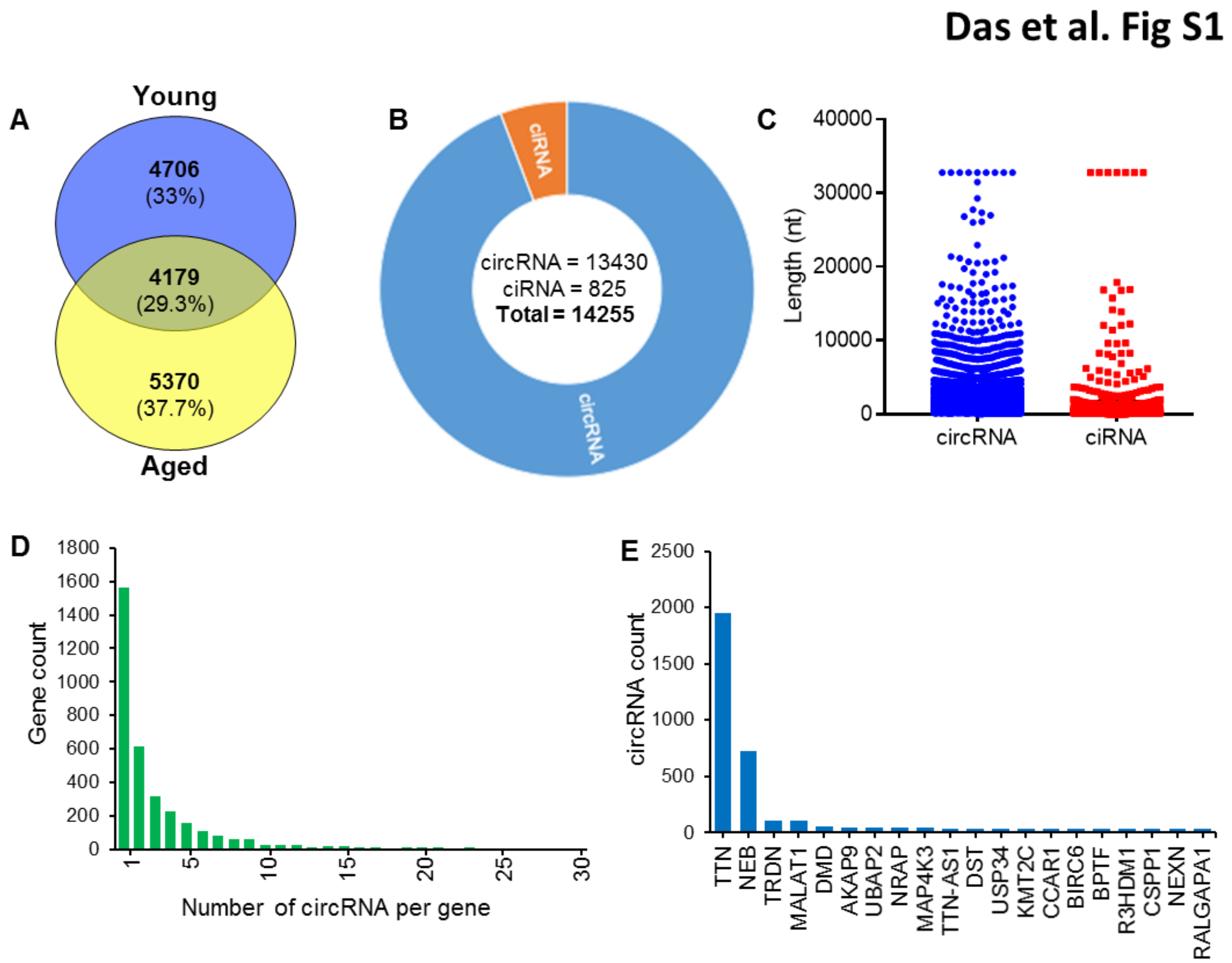

Supplementary Figure S1. CircRNA expression in aging human skeletal muscle. A. Venn diagram showing the number of circRNAs expressed in young and aged human skeletal muscle samples identified using CIRCexplorer2. **B.** Number of circRNAs that are generated from exonic circRNA or intronic ciRNAs sequences. **C.** Distribution of the length of exonic circRNAs and intronic ciRNAs expressed in gastrocnemius muscle detected with CIRCexplorer2. **D.** Distribution of the number of genes generating a different number of circRNAs. **E.** Number of circRNAs generated by various genes.

Das et al. Fig S2

A

**Skeletal muscle from satellite cell samples**<br>GEO Accession No. **GSE78611**; Bio Project No. **PRJNA30709**				
**Sample type**	**Donor Age**	**GEO Sub-ID**	**Bio Sample ID**	**SRA Run ID**
Young-Satellite Cell	21	GSM2072469	SAMN04284394	SRR3192525
Aged-Satellite cell	64	GSM2072468	SAMN04284393	SRR3192524

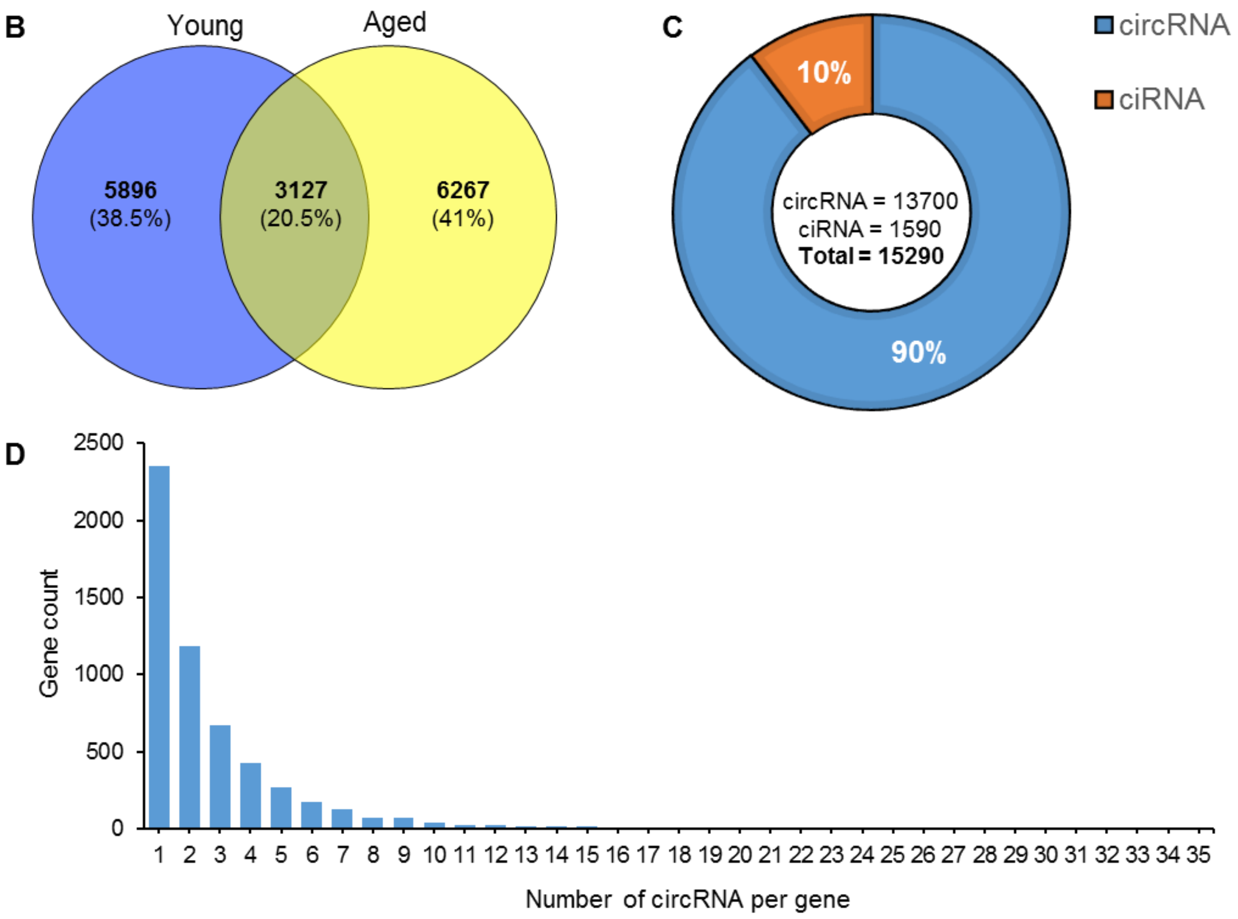

Supplementary Figure S2. CircRNA expression in human muscle satellite cells. A. NCBI GEO data sets used for circRNA analysis of young and aged human muscle satellite cells. **B.** Venn diagram showing the number of circRNAs expressed in young and aged human muscle satellite cells identified using CIRCexplorer2. **C.** Number of exonic circRNA or intronic ciRNAs expressed in human satellite cells. **D.** Number genes generating a different number of circRNAs in human muscle satellite cells.

Das et al. Fig S3

circCrebrf **(1216 nt) chr17|26739542|26743131|+**

CCCAGCGTAAGCGGAATGGACCCGCCTTTTGGGGATGCCTTTCGAAGCCACACCTTTTCAGAACAGACTCTGAT
GAGCACAGATCTCTTAGCCAACAGTTCTGATCCAGATTTCATGTATGAGCTGGATAGAGAGATGAATTATCAACA
GAATCCTAGAGACAACTTCCTTTCTTTGGAAGACTGCAAAGACATTGAAAATCTGGAGACTTTCACAGATGTCCT
GGACAATGAGGATGCTTTAACTTCAAACTGGGAACAGTGGGATACATACTGTGAAGACTTAACTAAGTACACGAA
GCTCACCAGCTGTGACATTTGGGGGACAAAAGAGGTGGATTACCTGGGTCTTGATGACTTTTCTAGCCCTTACC
AAGATGAAGAGGTCATCAGTAAAACTCCAACACTGGCCCAGCTCAATAGTGAGGACTCTCAGTCTGTTTCCGATT
CCCTTTATTATCCTGACTCACTCTTCAGTGTCAAACAAAATCCCTTGCCCCCCTCCTCTTTTCCTAGTAAAAAGAT
CACAAATAGAGCAGCTGCCCCTGTGTGTTCTTCAAAGACACTTCAGGCTGAGGTCCCATCATCAGACTGTGTCC
AAAAAGCAAGCAAACCTACTTCAAGCACACAGATCATGGTGAAGACCAACATGTATCATAATGAAAAGGTGAATT
TTCATGTTGAATGTAAAGACTATGTAAAAAAAGCAAAAGTCAAGATCAACCCTGTGCAACAGGGCCGGCCCTTGC
TGAGCCAGGTCCACATAGATGCAGCAAAGGAGAACACCTGCTACTGTGGAGCTGTGGCAAAGAGACAGGAGAG
AAGGGGGGTGGAGCCGCATCAGGGTCGGGGCACTCCTGCTTTGCCTTTCAAAGAAACCCAGGAGCTATTACTT
AGTCCTCTGACGCAGGATAGTCCTGGGTTGGTTGCCACAGCAGAGAGTGGCAGCCTTTCTGCCAGCACTTCTG
TTTCAGATTCATCCCAGAAAAAAGAAGAGCACAATTATTCTCTTTTTGTCTCTGACAACATGAGAGAACAGCCAAC
CAAATACAGTCCTGAAGATGATGAGGATGATGAAGATGAGTTTGATGATGAGGACCATGATGAAGGGTTTGGCA
GCGAGCATGAGCTTTCTGAAAATGAAGAGGAGGAAGAAGAGGAAGAGGATTATGAGGATGACAGAGATGATGA
TATCAGCGACACGTTCTCTGAACCAG

circPde4dip **(495 nt) chr3|97792738|97796808|-**

AACATCGAGCTGAAGGTTGAAGTGGAGAGCCTGAAACGAGAACTCCAGGACAGGAAACAGCATCTAGATAAAAC
ATGGGCCGATGCAGAGGATCTCAACAGCCAGAATGAGGCAGAGCTCCGGCGCCAGGTTGAAGAACGGCAGCA
GGAGACAGAACACGTTTATGAGCTCCTAGGGAACAAGATCCAGCTGCTGCAGGAGGAACCCAGGCTAGCAAAG
AATGAAGCCACAGAGATGGAGACTCTGGTGGAGGCAGAGAAGAGGTGCAATCTGGAGCTCTCAGAGAGGTGGA
CGAATGCTGCCAAGAACAGGGAAGATGCAGCAGGAGACCAGGAGAAGCCTGACCAATATTCTGAGGCACTGGC
TCAGAGGGACAGGAGAATTGAAGAGCTGAGGCAGAGCTTGGCTGCTCAGGAGGGGCTTGTGGAACAGCTGTCT
CAAGAGAAACGACAACTGTTACATCTGCTGGAGGAGCCAGCGAGCATGGAAGTGCAG

circRad52 **(303 nt) chr6|119920109|119921028|+**

CAGTACTTCCAGCCCCTCCAAAACACAGCACCCCTGTAACTGCAGCCTCAGAACTCCTCCAGGAGAAAGTCGTC
TTTCCAGATAACCTTGAAGAGAACCTTGAAATGTGGGACCTTACTCCAGACTTAGAGGACATCATTAAGCCCTTG
TGTAGAGCAGAACCAGCCCAAACTTCTGCCACTCGAACCTTCAACAACCAGGACAGCGTCCCACATATCCATTG
CCATCAGAAACCACAAGAAAAGCCTGGACCTGGGCACCTGCAGACCTGCAACACCAACCAGCATGTTCTAGGTA
GCAGAG

circMypn **(412 nt) chr10|63162221|63169430|-**

GTGGTATTGTGAAGGCAAGGAGCTTGAAAACTCTCCAGATATCCACATTGTGCAGGCAGGAAACCTTCACTCAC
TGACCATCGCCGAGGCCTTCGAAGAGGACACAGGACGATACTCATGCTTTGCTTCCAACATTTATGGGACAGAT
TCAACTTCCGCTGAGATTTACATAGAAGGAGTCTCTTCCTCTGACTCAGAAGGGGACCCTAACAAAGAAGAGAT
GAATCGAATCCAGAAGCCAAATGAGGTGTCCTCACCTCCCACCACCTCTGCAGCCATTCCTCCAGCAGCAGAAG
CCCAGCCTTTGGCGGCCCAGCCCAGAGTGTCCACCGTCCAGCAGTGTCAGAGTCCTACCAACTATTTGCAAGG
CCTGAACGGGAAGCCCATCATTGCAGCGCCAGTGTTTACGAAG

circZbtb20 **(511 nt) chr16|43569680|43577748|+**

ATGCAGCCACTCTCTGTCCCCTGCCCCAATGAACATCTGCACTAGGCCCAAGCCTTGGAGTGATTTACCTGAAG
AGTGACACCAATTATCTGGAAACTACTAAAGTATAATTGAGAGGAACAGGGTGAAGGAAATGCAAATGCGAAAG
GGAAGCAGTAGATGAGGAAACTGAAGCCCAGAGAAGTGAAGTGAGGTGCCCAAAGCCACACAGCAAGTTGCAG
GCACAGCTAGTTCCGTAGCTCAAGTCTCCTGACTCCCAGTCCAGTGTTCTTCCCATTACTCCACGGGTCCTGTC
TCTAAGCTTCCTGACAAATGCTAGAACGGAAGAAACCCAAGACAGCTGAAAACCAGAAGGCATCTGAGGAGAAT
GAGATTACTCAGCCGGGCGGATCCAGCGCCAAGCCGGCCCTTCCCTGCCTGAACTTTGAAGCTGTTTTGTCTC
CAGCCCCAGCCCTCATCCACTCGACACATTCACTGACAAACTCTCACGCTCACACCGGGTCATCTGATT

circNfix **(532nt) chr8|84771783|84772315|532|-**

GATGAGTTCCACCCGTTTATCGAGGCGCTGCTGCCTCACGTCCGAGCCTTCTCCTACACCTGGTTCAACCTGCA
GGCGCGGAAGCGCAAGTACTTCAAGAAGCACGAGAAGCGGATGTCAAAGGACGAGGAGCGCGCAGTGAAGGA
CGAGCTGCTGGGCGAGAAGCCTGAGATCAAGCAGAAGTGGGCATCCCGGCTGTTGGCCAAGCTGCGCAAAGA
CATCCGGCCCGAGTTCCGCGAGGACTTTGTGCTAACCATCACGGGCAAGAAGCCCCCCTGCTGCGTGCTTTCC
AACCCCGACCAGAAGGGCAAGATCCGGCGGATTGACTGCCTGCGCCAGGCTGACAAGGTGTGGCGGCTGGAC
CTGGTCATGGTGATTTTGTTTAAAGGGATCCCTTTGGAAAGTACTGATGGGGAGCGGCTCTACAAGTCGCCCCA
GTGCTCGAACCCCGGCCTGTGTGTCCAGCCACATCACATTGGAGTCACAATCAAAGAACTGGACCTTTATCTGG
CTTACTTTGTCCACACTCCGG

Supplementary Figure S3. Spliced sequences of validated circRNAs used for further analysis.

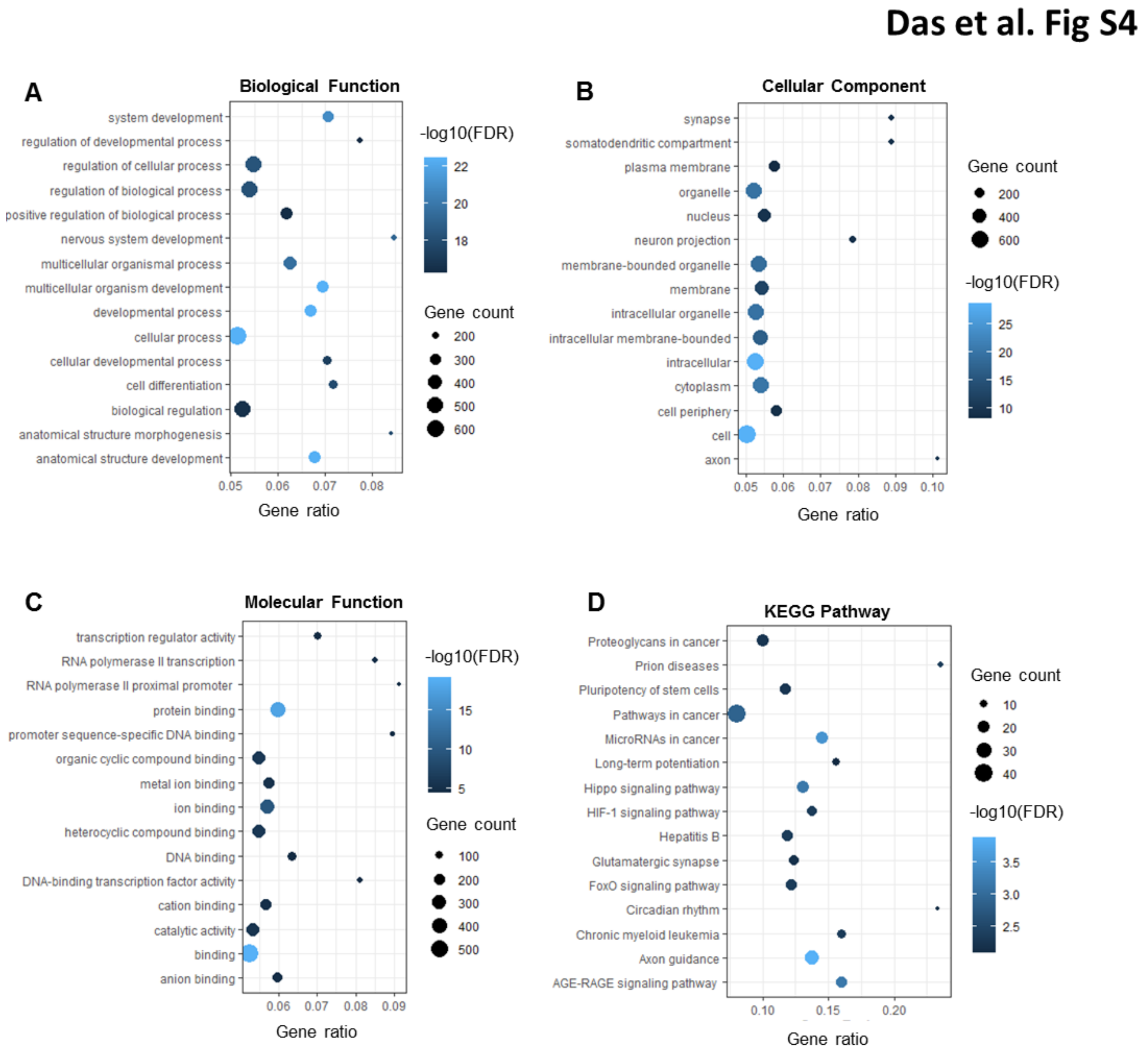

Supplementary Figure S4: Enrichment analysis of genes in the circRNA-miRNA-mRNA regulatory network. A-C. A bubble plot of the enriched GO terms for the biological process (A), cellular component (B), and molecular function (C) for the genes in the circRNA-miRNA-mRNA regulatory network. **D.** A bubble plot of the enriched KEGG pathways of genes associated with the circRNA-miRNA-mRNA regulatory network.

Das et al. Fig S5

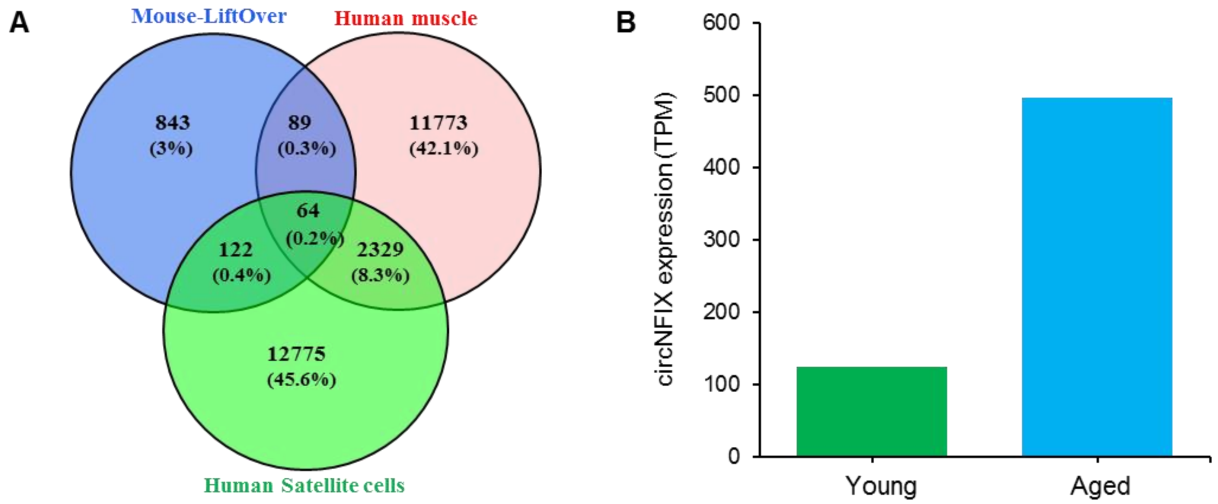

C ***circNFIX*; chr19|13025020|13025552|+; NM_001365902; 532 nt**

GATGAGTTCCACCCGTTCATCGAGGCACTGCTGCCTCACGTCCGCGCTTTCTCCTACACCTGGTTCAACCTGCAGGCGCGGAAGC
GCAAGTACTTCAAGAAGCATGAAAAGCGGATGTCGAAGGACGAGGAGCGGGCGGTGAAGGACGAGCTGCTGGGCGAGAAGCCC
GAGATCAAGCAGAAGTGGGCATCCCGGCTGCTGGCCAAGCTGCGCAAGGACATCCGGCCCGAGTTCCGCGAGGACTTCGTGCTG
ACCATCACGGGCAAGAAGCCCCCCTGCTGCGTGCTCTCCAACCCCGACCAGAAGGGCAAGATCCGGCGGATTGACTGCCTGCGC
CAGGCTGACAAGGTGTGGCGGCTGGACCTGGTCATGGTGATTTTGTTTAAGGGGATCCCCCTGGAAAGTACTGATGGGGAGCGG
CTCTACAAGTCGCCTCAGTGCTCGAACCCCGGCCTGTGCGTCCAGCCACATCACATTGGAGTCACAATCAAAGAACTGGATCTTTA
TCTGGCTTACTTTGTCCACACTCCGG

D

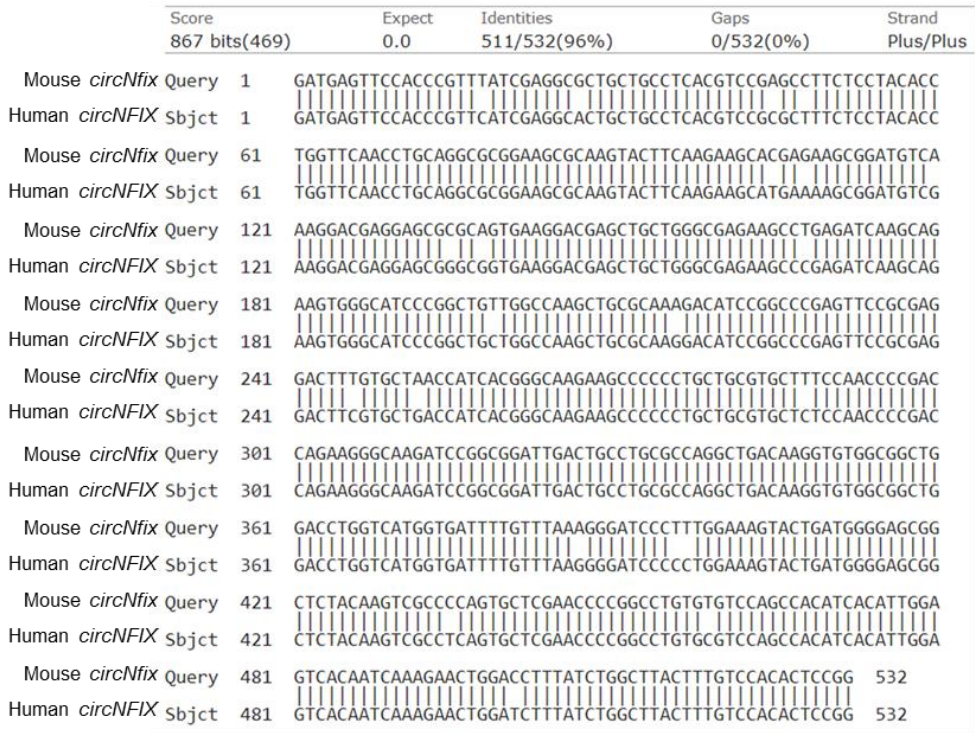

Supplementary Figure S5. **A.** Venn diagram of mouse circRNAs and human muscle tissue and satellite cells. **B.** Expression levels of *circNFIX* in young and aged satellite cells. **C.** Sequence of human *circNFIX*. **D.** NCBI BLAST results for sequence homology between mouse *circNfix* and human *circNFIX*.

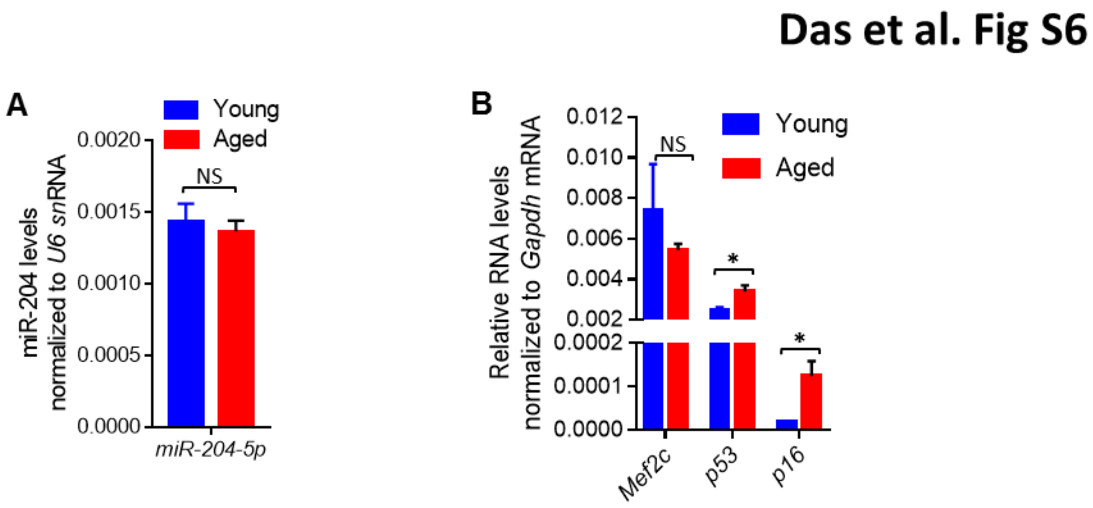

Supplementary Figure S6: Expression analysis of miR-204 and Mef2c mRNA in aging skeletal muscle of mice. A. RT-qPCR analysis of *miR-204* in young and aged gastrocnemius skeletal muscle of mice. **B.** RT-qPCR analysis of *Mef2c* in young and aged mouse gastrocnemius skeletal muscle.

Das et al. Fig S7

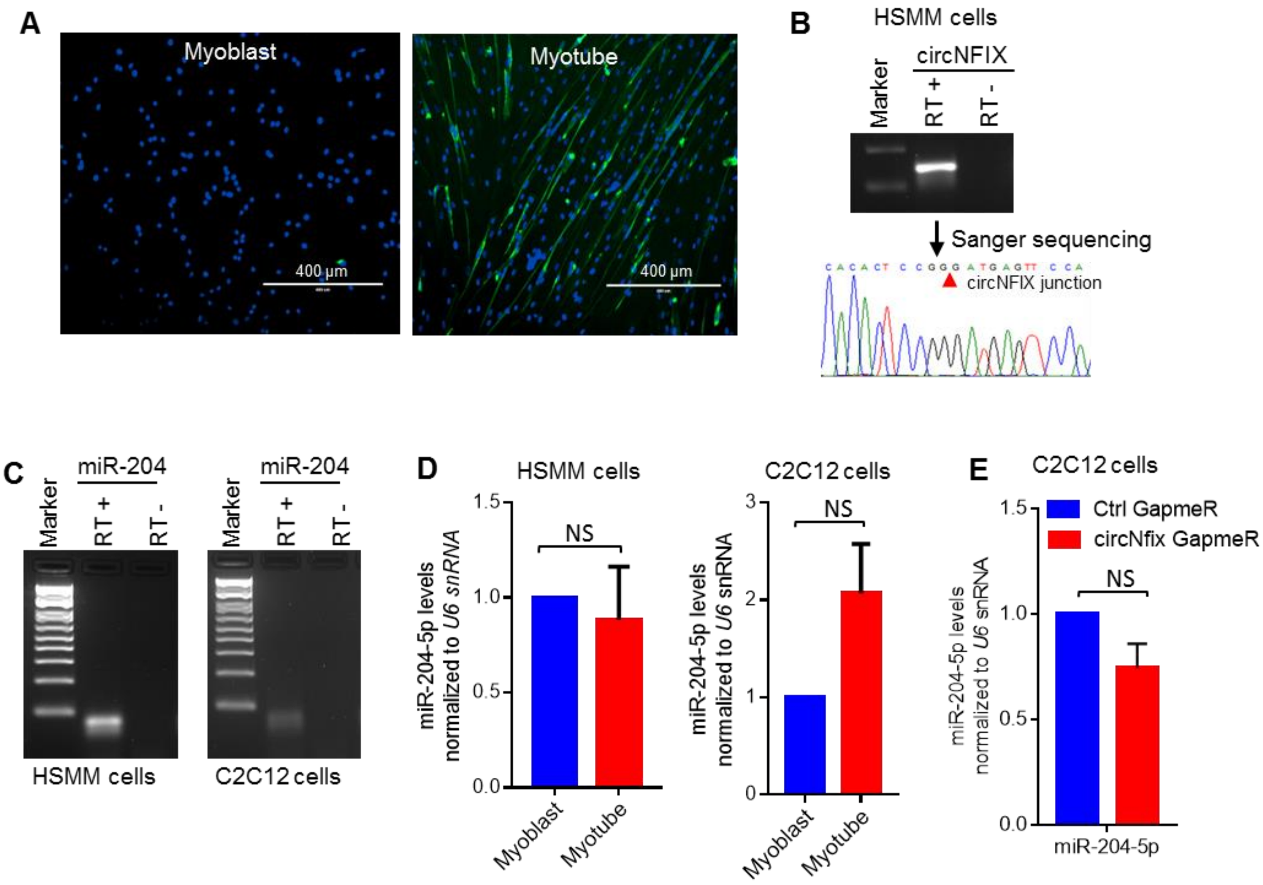

Supplementary Figure S7: Expression analysis of *circNfix* and miR-204. A. Fluorescent images of proliferating and four-day differentiated primary HSMM cells with MYH immunostaining. The blue nuclei are stained with DAPI. **B.** RT-PCR analysis of *circNFIX* using divergent primers in HSMM cells and visualized in an SYBR Gold stained 2% agarose gel (*top*). The bottom panel shows the Sanger sequencing of the purified circNFIX PCR product confirming the amplification of the backsplice junction sequence (red arrowhead). **C.** The RT-PCR products of miR-204 from HSMM and C2C12 cells resolved and visualized in SYBR Gold stained 2% agarose gel. **D.** RT-qPCR analysis of *miR-204* in proliferating myoblasts and four-day differentiated myotubes of HSMM and C2C12 cells. **E.** RT-qPCR analysis of miR-204-5p levels in C2C12 after silencing of circNfix using GapmeR. Data in D and E are the means ±SEM from three independent experiments. *, $p<0.05$.

SUPPLEMENTARY FIGURES AND TABLES

Supplementary Table S1: circRNA expression analysis of young and old mouse skeletal muscle samples using CIRCexplorer2

Supplementary Table S2: RNA-seq data for young and aged human skeletal muscle samples used in the study

Supplementary Table S3: circRNA expression analysis of young and old human skeletal muscle samples using CIRCexplorer2.

Supplementary Table S4: circRNA expression analysis of young and old human muscle satellite cells using CIRCexplorer2.

Supplementary Table S5: Oligo sequences used in the study

Supplementary Table S6: CircRNA-miRNA-mRNA regulatory network of selected circRNAs in mouse skeletal muscle.

Supplementary Table S7: GO and KEGG analysis of target genes in circRNA-miRNA-mRNA regulatory network
